# Severe presentation and complex brain malformations in an individual carrying a *CCND2* variant

**DOI:** 10.1002/mgg3.708

**Published:** 2019-05-06

**Authors:** Gerarda Cappuccio, Lorenzo Ugga, Elena Parrini, Alessandra D’Amico, Nicola Brunetti‐Pierri

**Affiliations:** ^1^ Department of Translational Medicine Federico II University Naples Italy; ^2^ Telethon Institute of Genetics and Medicine Pozzuoli, Naples Italy; ^3^ Department of Advanced Biomedical Sciences Federico II University Naples Italy; ^4^ Pediatric Neurology, Neurogenetics and Neurobiology Unit and Laboratories, Neuroscience Department, A. Meyer Children's Hospital University of Florence Florence Italy

**Keywords:** CCND2, Megalencephaly‐polymicrogyria‐polydactyly‐hydrocephalus, MPPH

## Abstract

**Background:**

Megalencephaly‐polymicrogyria‐polydactyly‐hydrocephalus (MPPH) is a developmental brain disorder characterized by megalencephaly and bilateral perisylvian polymicrogyria due to defects in genes of the PI3K‐AKT pathway. Only a few patients with *CCND2* mutations have been reported to date.

**Methods:**

We describe an individual harboring a de novo variant in *CCND2* undergoing neuroradiological evaluation including diffusion tensor imaging (DTI).

**Results:**

The individual presented with a severe brain malformation extending to both brainstem and cerebellum with hypomyelination not previously reported in *CCND2*‐related disorder. Severe hypoplasia and white matter disorganization were confirmed by DTI.

**Conclusion:**

This report expands the phenotypic spectrum of the disorder due to *CCND2* variants.

## 1 INTRODUCTION

Megalencephaly‐polymicrogyria‐polydactyly‐hydrocephalus (MPPH) syndrome is a disorder of brain overgrowth (megalencephaly [MEG]) with a recurrent pattern of physical and neuroimaging anomalies. MPPH is largely caused by de novo mutations in genes of PI3K‐AKT pathway. Variants in *CCND2* (OMIM 123833) encoding cyclin D2, a downstream mediator of PI3K‐AKT pathway, have been reported in 14 patients with MPPH so far (Maini et al., 2018; McDermott et al., 2018; Mirzaa et al., 2014). Recurrent variants at four different codons (p.Lys270, p.Thr280, p.Pro281 and p.Val284) have been reported (Maini et al., 2018; McDermott et al., 2018; Mirzaa et al., 2014). Neuroimaging of MPPH due to *CCND2* variants includes progressive and striking MEG and polymicrogyria (PMG) without vascular anomalies (Mirzaa, Riviere, & Dobyns, 2013). Here, we report a 20‐year‐old individual with a de novo *CCND2* variant presenting with a previously unreported complex pattern of cerebral, cerebellar, and brainstem malformations.

## 2 CLINICAL REPORT

The proband is a male individual who was the second child of unrelated parents. He was born after a full‐term pregnancy by caesarean section with a birth weight of 3,780 grams and an occipito‐frontal circumference (OFC) of 38.5 cm (+3SD). Prenatal ultrasound at 30 weeks of gestation revealed enlarged cerebral ventricles and polyhydramnios. He had no perinatal issues but was found to have bilateral postaxial polydactyly of the hands that was surgically corrected at 3 years of age.

By the age of 7 months, he developed infantile spasms partially responsive to ACTH and vigabatrin. Starting from the age of 2 years, he developed drug‐resistant tonic‐clonic generalized seizures. Currently, he is on valproate and felbamate and shows clusters of two or three seizures per day. EEG showed abnormal background activity and centro‐temporal spikes with secondary generalization.

His development was severely delayed without regression and at his last examination performed when he was 20 years old, he had social smiling and head control, but he could neither walk nor sit independently and was wheelchair‐bound. He did not have purposeful hand movements and was unable to speak meaningful words. His weight was 31 Kg (<5th centile), length 170 cm (10th–25th centile) and OFC was 63.7 cm (+4SD). He had frontal bossing.

**Figure 1:**
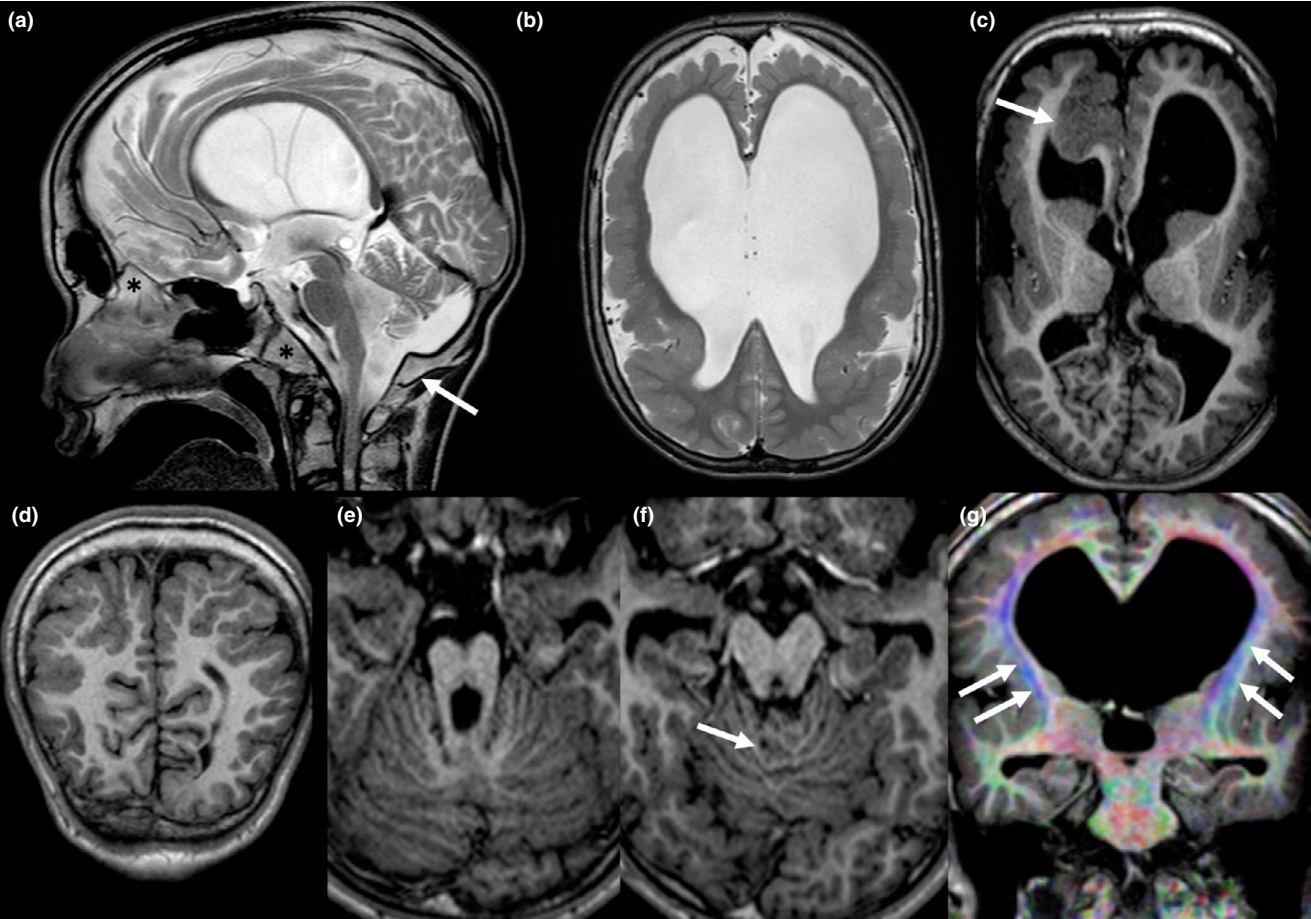
Sagittal turbo spin‐echo T2‐weighted sequence (a) showing macrocrania, dysmorphic skull base, anti‐clockwise rotation and hypoplasia of the vermis, brainstem hypoplasia, and stretched corpus callosum; a dysmorphic skull base with thickened clivus, *crista galli* and posterior margin of the foramen magnum (asterix and arrow, respectively) is also evident. Bilateral polymicrogyria and ventriculomegaly are shown on axial turbo spin‐echo T2‐weighted image (b). Axial reformation of 3D turbo field echo T1 weighted sequence showing dysmorphic basal ganglia and pronounced infolding and thickening of the right frontal cortex (arrow) (c). Coronal reformation of 3D turbo field echo T1 weighted sequence showing parieto‐occipital polymicrogyria (d). Dysmorphic midbrain and disorganized foliation [arrow on (f)] on axial reformation of 3D turbo field echo T1 weighted sequence (e and f). Coronal reformats of colored Fractional Anisotropy map from Diffusion Tensor Imaging showing white matter hypoplasia and fascicle abnormalities, including laterally displaced cortico‐spinal tracts (arrows) and absence of superior longitudinal fasciculus (g)

He showed patent foramen ovale and ductus arteriosus evolving into spontaneous closure. An abdomen ultrasound did not detect any abnormalities, but he had bilateral cryptorchidism that was surgically corrected. At the age of 17 years a melanocytic nevus was surgically removed. He had feeding difficulties requiring pureed diet and bilateral hip displacement. A 1.5T brain MRI scan at the age of 19 years confirmed the macrocephaly and showed dysmorphic appearance of the skull base characterized by thickening of the clivus, *crista galli*, and posterior foramen magnum margin (Figure 1a). Brain MRI showed extensive bilateral PMG with thick, irregular, and coarse appearance of the cortical surface of cerebral hemispheres partially sparing the temporo‐occipital regions with infolding and marked thickening of polymicrogyric cortex of the right mesial frontal lobe (Figure 1b–d). White matter volume was widely and severely reduced and the corpus callosum was thinned, stretched, and arched‐shape (Figure 1a,b). Other midline abnormalities included fenestration of the septum pellucidum and hypoplasia of the anterior and posterior commissures. Moreover, hippocampal hypoplasia and malrotation was observed. The supratentorial ventricular system was enlarged and the thalami and lentiform nuclei were hypoplastic. In posterior cranial fossa, an anti‐clockwise rotation of the vermis that was hypoplastic and with disorganized foliation and abnormal orientation of sulci was noted (Figure 1e,f). Hypoplasia of the brainstem and particularly of the pons was observed whereas superior cerebellar peduncles were thickened and horizontalized although a typical “molar tooth” sign was not observed (Figure 1a,e,f). White matter hypoplasia was confirmed by diffusion tensor imaging that also showed multiple fascicle abnormalities (Figure 1g).

Genetic testing was performed as part of clinical management after obtaining parental informed consent. Chromosome microarray analysis did not detect pathogenic chromosomal rearrangements. A targeted next‐generation sequencing of a panel of 182 genes associated with brain malformations performed using a SureSelect custom capture (Agilent Technologies, Santa Clara, CA) revealed an heterozygous *CCND2* variant NM_001759.3: c.839C>T, p.(Thr280Ile). This variant was confirmed by Sanger sequencing in the proband and was absent in both parents.

## 3 DISCUSSION

Brain MRI abnormalities detected so far in individuals harboring *CCND2* variants include hydrocephalus, ventriculomegaly, and PMG mainly in the perisylvian region (Maini et al., 2018; McDermott et al., 2018; Mirzaa et al., 2014). The Thr280 residue was previously found to be affected in individuals with PMG (McDermott et al., 2018; Mirzaa et al., 2014). Moreover, a 10‐year‐old female individual was previously reported to carry the same p.(Thr280Ile) variant detected in the individual herein described, and presented with a largely overlapping phenotype characterized by severe developmental delay and drug‐resistant epilepsy, generalized MEG, widespread PMG, hydrocephalus requiring surgical intervention, corpus callosum hypoplasia, and postaxial polydactyly (Garavelli et al., 2007; Maini et al., 2018). However, the individual herein described did not require shunting for the hydrocephalus, and presented with additional and previously unreported brain findings including skull base abnormalities, hippocampus hypoplasia and malrotation, hypomyelination, and cerebellar anomalies. Moreover, brainstem hypoplasia and dysmorphism of the midbrain were also noted and these features have not been previously reported in cases harboring *CCND2* defects.

In conclusion, we report a further patient with a *CCND2* variant showing complex pattern of cerebral, cerebellar and brainstem malformations that expand the spectrum of brain abnormalities due to *CCND2* defects.

## CONFLICT OF INTEREST

The authors declare no competing financial interests.

## AUTHORS’ CONTRIBUTIONS

G. Cappuccio and N. Brunetti‐Pierri evaluated the patient. A. D'Amico and L. Ugga performed and interpreted the brain MRI. E. Parrini performed and interpreted the genetic testing.
